# Drug induced hyperpigmentation: systematic review and meta-analysis

**DOI:** 10.3389/fmed.2026.1674278

**Published:** 2026-04-07

**Authors:** Ruaa Alharithy, Kayan Alotaibi, Rawan Bin Salamah, Reem Altamimi, Norah Alqntash, Reem Alsarhan, Leen Khosyfan, Shahad Alrowais

**Affiliations:** 1 Security Forces Hospital, Riyadh, Saudi Arabia; 2 Collage of medicine, Princess Nourah Bint Abdulrahman University, Riyadh, Saudi Arabia; 3 College of Medicine, Princess Nourah Bint Abdulrahman University, Riyadh, Saudi Arabia

**Keywords:** 5-fluorouracil, adverse drug reaction, antimalarial agents, cutaneous side effects, dermatology, drug-induced hyperpigmentation, hydroxychloroquine, meta-analysis

## Abstract

**Background:**

Drug-induced hyperpigmentation (DIH) represents a significant subset of acquired pigmentation disorders and poses diagnostic challenges due to delayed onset and polypharmacy. This systematic review and meta-analysis aimed to identify medications significantly associated with DIH and evaluate their reported incidence.

**Methods:**

A systematic search was conducted across PubMed, Scopus, Web of Science, and Cochrane Library for studies published between 2002 and June 2024. Eligible studies reported DIH as an outcome with incidence or descriptive data. Pooled proportions were calculated using a random-effects model, and heterogeneity was assessed via the I^2^ statistic.

**Results:**

Twenty-two studies met the inclusion criteria. The overall pooled incidence of DIH was 36.7% (95% CI: 0.291–0.444). Subgroup analyses revealed the highest incidences with tyrosine kinase inhibitors (89.2%) and MC4R agonists (71.4%), followed by antibiotics (52.0%), antineoplastic agents (35.5%), and antimalarials (29.0%). Commonly implicated agents included minocycline, hydroxychloroquine, and hydroxyurea.

**Conclusion:**

DIH is a prevalent adverse drug reaction with considerable variation in incidence across drug classes. Recognition of high-risk medications is essential for prompt diagnosis and clinical management.

**Systematic review registration:**

The study protocol was pre-registered in the International Prospective Register of Systematic Reviews (PROSPERO: CRD42024529250).

## Introduction

Drug-induced hyperpigmentation (DIH) is a frequent concern in dermatology, accounting for up to 20% of all cases of acquired pigmentation (1). It manifests as darkening of the skin that can occur in various locations, including sun-exposed areas and mucous membranes; the discoloration is acquired and usually grows slowly and spreads insidiously, worsening over months or years after initiation of the offending drug (2). DIH can be caused by a wide range of drugs, including non-steroidal anti-inflammatory drugs (NSAIDs), antimalarial drugs, amiodarone, cytostatic agents, tetracycline, and psychopharmaceuticals. However, confirming true drug associations can be challenging, especially when pigmentation onset is delayed, and there is coexisting polypharmacy (1). This complexity underscores the importance of thorough medical histories and dermatological evaluations to pinpoint the causative agent accurately. Furthermore, factors such as individual patient susceptibility, genetic predisposition, and environmental influences may also contribute to the development of DIH.

DIH can be a significant cosmetic concern, potentially impacting a patient’s quality of life. Due to the visible nature of the pigmentation changes, patients may experience emotional distress, self-consciousness, and social withdrawal (3). Therefore, effective management strategies for DIH should focus on treating the pigmentation itself and addressing the condition’s emotional and psychological aspects. Understanding DIH remains challenging, often relying on case reports and series with limited high-quality studies to confirm cause-and-effect relationships. In 2025, revisiting the topic of DIH is particularly important for several reasons. First, the therapeutic landscape has expanded with the introduction of novel drug classes, including tyrosine kinase inhibitors and melanocortin-4 receptor (MC4R) agonists, both of which are strongly linked to pigmentation changes and were not widely represented in earlier reviews. Second, environmental factors such as global climate change and associated increases in ultraviolet (UV) radiation exposure may amplify the risk and severity of DIH, especially in sun-exposed populations. Third, recent trends in chronic disease management, including long-term use of antimalarials, immunomodulators, and targeted anticancer therapies, have increased the frequency and visibility of pigmentation-related adverse events. Collectively, these developments underscore the need for an updated systematic review and meta-analysis to provide clinicians with contemporary evidence, guide risk stratification, and improve management strategies for affected patients. This systematic review and meta analysis aimed to identify the medications that have a significant association with skin hyperpigmentation in order to provide a better understanding of risk factors, diagnosis, and management of DIH.

## Methods

We conducted this systematic review and meta-analysis in compliance with the Preferred Reporting Items for Systematic Reviews and Meta-Analyses (PRISMA) guidelines. The study protocol was pre-registered in the International Prospective Register of Systematic Reviews (PROSPERO: CRD42024529250).

### Search strategy

To ensure a comprehensive search, four databases were utilized: PubMed, Scopus, Web of Science, and Cochrane Library. The search was limited to studies published between January 2002 and June 2024. The search strategy included the following keywords: (“drug-induced pigmentation” OR “hyperpigmentation” OR “cutaneous discoloration” OR “drug eruption” OR “adverse drug reaction” OR “skin pigmentation”). Boolean operators such as “AND” and “OR” were used to combine terms and broaden the search scope. Reference lists of included articles and related reviews were also screened manually to capture any additional eligible studies.

### Inclusion criteria

Studies were included if they met the following criteria: original research articles published in English, conducted in human subjects, and investigating drug-induced hyperpigmentation either descriptively or quantitatively. Studies that reported the incidence, prevalence, type, or characteristics of hyperpigmentation following drug exposure were eligible. Exclusion criteria included review articles, editorials, case reports, non-English articles without an English summary, and studies with unavailable full texts. For the purposes of this review, hyperpigmentation was defined as any acquired, drug-associated darkening or discoloration of the skin, mucous membranes, nails, hair, or ocular structures, as documented clinically by the study authors. Cases were included if pigmentation was persistent beyond the expected course of transient erythema or inflammation and was attributed to pharmacological exposure. We excluded reports of cutaneous reactions where pigmentation was secondary to inflammatory dermatoses (e.g., post-inflammatory hyperpigmentation following drug eruptions), fixed drug eruptions, or other unrelated pigmentary disorders, as these represent distinct pathophysiological processes. This restriction ensured that our analysis focused specifically on primary drug-induced hyperpigmentation rather than pigmentation secondary to other cutaneous reactions.

### Study selection

After conducting the search, all retrieved articles were imported into Rayyan software for de-duplication. Title and abstract screening were conducted independently by two authors. Any conflicts were resolved through discussion with a third author. Full texts of potentially eligible studies were assessed against the inclusion and exclusion criteria. Studies that fulfilled the criteria were included in the final analysis.

### Data extraction

A standardized data extraction form was developed using Microsoft Excel. All reviewers independently extracted the following data from the included studies: name of first author, year of publication, country, study design, sample size, patient demographics, drug class and name, dosage, treatment duration, anatomical site and type of hyperpigmentation, number of affected individuals. Discrepancies in data extraction were resolved by discussion and consensus.

### Risk of bias assessment

The risk of bias of the included studies was assessed using tools appropriate to the study design. Observational studies were evaluated using the Newcastle-Ottawa Scale (NOS), randomized controlled trials were assessed using the Cochrane Risk of Bias tool. Two reviewers conducted the assessments independently, and any disagreements were resolved by a third reviewer.

### Data analysis

Meta-analyses were conducted using a random-effects model. The primary outcome was the pooled proportion of patients who developed drug-induced hyperpigmentation. Subgroup analyses were performed according to drug classes to assess variation across pharmacological groups. Heterogeneity among studies was assessed using the I^2^ statistic, interpreted as follows: 0–50% (low heterogeneity), 50–75% (moderate heterogeneity), and >75% (high heterogeneity). Sensitivity analyses were also performed to examine the robustness of the findings. The restricted maximum-likelihood (REML) estimator was applied for all random-effects models. To allow for the inclusion of studies with zero reported events, a continuity correction of 0.5 was added to all cells of applicable studies prior to analysis.

## Results

### Study selection

**Figure 1:**
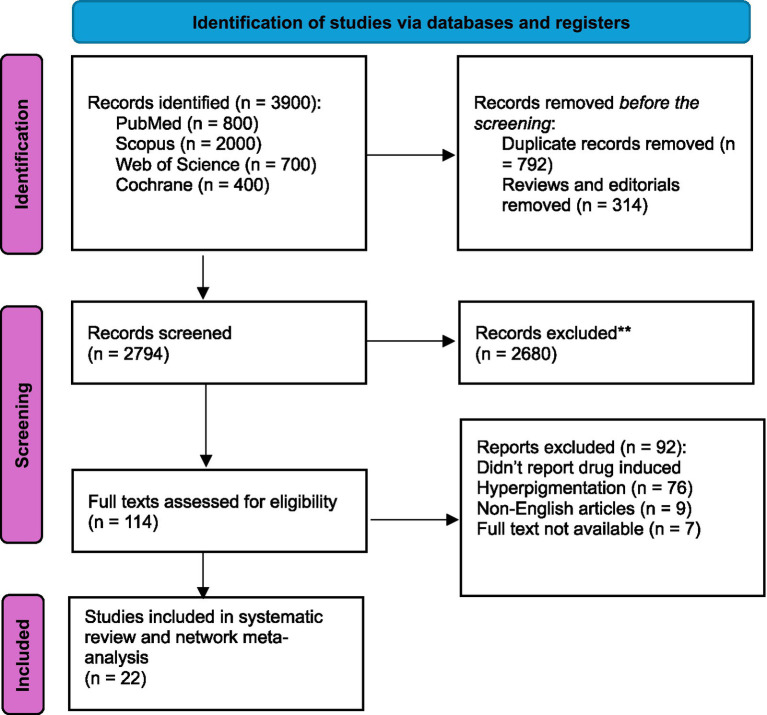
PRISMA chart summarizing the flow of the studies selection.

Our initial database search yielded 3,900 potential articles. We then removed 1,106 records prior to the screening phase: 792 were duplicates and 314 were either reviews or editorials. This resulted in 2794 articles that underwent title and abstract screening. This stage led to the exclusion of (2680) articles, leaving (114) articles that warranted full-text review to assess eligibility. We excluded (92) articles that did not verify our inclusion criteria. Ultimately, we identified (4) studies as meeting all criteria to be included in the systematic review and meta-analysis. An overview of this entire selection process is depicted in Figure 1.

### General characteristics

Table 1 details 22 studies examining drug-induced hyperpigmentation across diverse pharmacological categories. The included studies utilized a range of methodologies including prospective studies, RCTs, Phase II and III trials, retrospective analyses, and cross-sectional surveys. Anti-neoplastic agents, such as topical and intralesional 5-fluorouracil, alongside systemic regimens involving S-1 combined with raltitrexed or oxaliplatin, were assessed in the context of conditions like vitiligo and cancer. Cytotoxic agents were represented by intralesional bleomycin, explored for its use in managing vascular malformations and keloids. The anti-glaucoma class, primarily topical latanoprost and its combinations, was associated with hyperpigmentation in the iris and eyelid. Antimalarial agents, notably hydroxychloroquine, were linked to hyperpigmentation at various locations throughout the body. Antibiotics, such as minocycline and clofazimine, were examined in relation to cutaneous manifestations. Immunomodulatory agents, including topical corticosteroids and systemic interferon alfa, are also included, as is the tyrosine kinase inhibitor imatinib mesylate, and the MC4R agonist, setmelanotide; all of which were observed to be associated with different types of hyperpigmentation. The primary site of hyperpigmentation was the skin, but involvement of the iris, eyelids, oral mucosa, nails, and gums were also recorded.

**Table 1:** The general characteristics of the included studies.

Author and year	Study design	Country	Treatment group/s (sample size)	Dose	Localization of hyperpigmentation	Drug indication	Follow-up period/s
Anti-neoplastic agents							
Mina 2018 (39)	Prospective study	Egypt	Topical 5-fluorouracil group (*n* = 25) Topical Tacrolimus group (*n* = 25)	250 mg/5 mL on 15 g liposomal base once daily for 2 weeks over 1 patch and topical tacrolimus ointment over the other patch once daily for 2 weeks. This procedure was repeated every 2 weeks for every patient for maximum 6 months (12 sessions)	Cutaneous	vitiligo	Three months after the last session
Chen 2019 (41)	Phase II Trial	China	Systemic S-1 plus Raltitrexed (*n* = 46)	Oral S-1 (80–120 mg for 14 days every 3 weeks) plus an intravenous infusion of raltitrexed (3 mg/m2 on day 1 every 3 weeks)	NR	Refractory metastatic colorectal cancer	every 12 weeks from the time patients stopped treatment
Ando 2020 (43)	Phase II Trial	Japan	Systemic S-1 plus oxaliplatin (*n* = 88)	Oral S-1 (40–60 mg twice daily on days 1–14 every 3 weeks) plus intravenous oxaliplatin (130 mg/m2 on day 1 for eight courses)	Cutaneous	stage III colon cancer	Three years
Alhamzawi 2021 (44)	Prospective study	Iraq	Intralesional 5-Fluorouracil (*n* = 24)	1 mL/cm2/keloid of 5-FU (50 mg/mL)	Cutaneous	Keloids management	Two months, four months, and six months of the intervention
Abdou 2022 (47)	Prospective study	Egypt	Topical 5-fluorouracil (*n* = 24)	Vial of 500 mg/10 mL every 2 weeks for 6 sessions	Cutaneous	vitiligo	During treatment period
Wang 2022 (4)	Phase II Trial	China	Mitoxantrone hydrochloride liposome (Lipo-MIT) (*n* = 30) Mitoxantrone hydrochloride (MIT) injection (*n* = 30)	20 mg/m^2^ of Lipo-MIT or 14 mg/m^2^ of MIT intravenously every 28 days, for up to 8 cycles until disease progression, intolerable toxicity, or death	Cutaneous	Advanced breast cancer	During treatment period
Abhirami 2024 (48)	Prospective study	India	Topical 5-fluorouracil (*n* = 30)	5% 5-Fluorouracil was applied on microneedled patch.	Cutaneous	vitiligo	During treatment period
Cytotoxic agents							
Mohan 2015 (50)	Retrospective study	South Africa	Intralesional bleomycin injection (*n* = 32)	0.5 mg/ kg per dose and the volume was estimated by the size of the lesion	Cutaneous	Low flow vascular malformations	Mean follow-up of 38 months
Bekkers 2024 (9)	RCT	Netherlands	Intralesional bleomycin (*n* = 14)	One USP/ml delivered to the lesion, with a maximum dose of 2 USP bleomycin per treatment (administered three times with times space of 28 days between each time)	Cutaneous	Keloids management	during treatment and four weeks after the end of treatment
Anti-glaucoma agents							
Teus 2002 (40)	Prospective cohort study	Spain	Topical latanoprost (n = 43)	NR	Iris	Glaucoma	A minimum of 12 month
Goldberg 2008 (12)	RCT	Australia	Topical latanoprost (*n* = 4,638)	NR	Iris	Glaucoma	Every 6 months for five years
Alm 2008 (17)	Phase IIIb Trial	USA	Latanoprost/Timolol (*n* = 974) adjunctive latanoprost (*n* = 380)	NR	Iris	Glaucoma	During treatment for five years
Nakakura 2012 (11)	RCT	Japan	latanoprost/timolol+ brinzolamide (*n* = 20) Dorzolamide/timolol+ latanoprost (*n* = 16)	latanoprost 0.005%/timolol 0.5% + brinzolamide 1% Dorzolamide 1%/timolol 0.5% + latanoprost 0.005%	Eyelid	Glaucoma	Four weeks and 12 weeks after the start of treatment
Antimalarial agents							
Skare 2011 (5)	Cross-sectional study	Brazil	Antimalarial agents (*n* = 209)	NR	Cutaneous, cheeks, hard palate, diffuse mucous membrane, nails and hair	Malaria	–
Bahloul 2017 (6)	Cross-sectional study	Tunisia	Systemic hydroxychloroquine (*n* = 41)	NR	Cutaneous, lips, gum	systemic lupus erythematosus (n = 30), dermatomyositis (n = 5), rheumatoid arthritis (n = 4), lichen actinicus (n = 1) and sarcoidosis (n = 1)	–
Yin 2024 (7)	Cross-sectional study	China	Systemic hydroxychloroquine (*n* = 316)	NR	Cutaneous, lips, nail	systemic lupus erythematosus (n = 214), Sjogren’s syndrome (n = 50), rheumatoid arthritis (n = 15) and others (n = 37)	–
Antibiotics agents							
Hanada 2016 (49)	Retrospective study	USA	Systemic Minocycline (*n* = 291)	NR	Cutaneous	orthopedic infections	A mean of 4.8 years
Tang 2015 (13)	RCT	China	systemic clofazimine (*n* = 53) Other anti-tuberculous drugs (*n* = 52)	Clofazimine: 100 mg per day for 21 months.	Cutaneous	Multidrug Resistant Tuberculosis	During treatment
Immunomodulators agents							
Ah 2019 (42)	Prospective cohort study	South Korea	Topical corticosteroid (*n* = 1,103)	NR	Cutaneous	Allergic contact dermatitis (n = 304), Urticaria (n = 204), Atopic dermatitis (n = 191), Pruritus (n = 123), Other dermatitis (n = 120) and Others (n = 250)	One, two and six months after study enrolment.
Tsilika 2013 (46)	Prospective study	France	Systemic interferon alfa (*n* = 77)	For 3 months	Cutaneous, nail, gum, the hard palate, and the tongue	Hepatitis C	During treatment
Tyrosine kinase inhibitors							
	Cross-sectional study	Brazil	Systemic imatinib mesylate (*n* = 74)	NR	Oral mucosa	leukemia	–
MC4R agonist							
Clément 2020 (10)	Phase III Trial	Canada, USA, Belgium, France, Germany, the Netherlands, and UK	Systemic setmelanotide	Individualized therapeutic dose, defined as weight loss of approximately 2–3 kg per week for adults or approximately 1–2 kg per week for paediatric participants, up to a maximum dose of 3 mg	Cutaneous	Obesity	During treatment for one year

NR: not reported; RCT: Randomized controlled trial; USP/ml: United States Pharmacopeia units per milliliter.

### Baseline characteristics

Table 2 summarizes the baseline characteristics and hyperpigmentation data from the included studies. The mean patient age across studies ranged from 21 to 71.9 years. While some studies included only female participants, the proportion of male participants reached up to 69.8% in certain cohorts. Notably, the incidence of cutaneous pigmentation was reported as 100% in most studies that assessed this outcome, with a few exceptions. Among studies investigating antimalarial agents, the prevalence of cutaneous pigmentation ranged from 33 to 95.2%. Mucosal pigmentation was largely absent in most study populations, except in studies examining tyrosine kinase inhibitors, where it was reported in 100% of cases, and in those receiving antimalarial agents, where it ranged from 4.8 to 33.3%.

**Table 2:** Baseline and hyperpigmentation data from the included studies.

Author and year	Group/s	Age (years), mean ± SD	Males, *n* (%)	Cutaneous pigmentation, *n* (%)	Muscous membrane pigmentation, *n* (%) *
Anti-neoplastic agents					
Mina 2018 (39)	Total sample	26.4 ± 15.3	10 (40)	4 (100%)	0 (0)
Chen 2019 (41)	Total sample	57 ± 12.2	22 (47.8)	NR	NR
Ando 2020 (43)	Total sample	64 ± 6.7	47 (53.4)	43 (100)	0 (0)
Alhamzawi 2021 (44)	Total sample	24.3 ± 10.5	14 (58.4)	20 (100)	0 (0)
Abdou 2022 (47)	Total sample	25.6 ± 18.7	6 (30)	4 (100)	0 (0)
Wang 2022 (4)	Lip-MIT group	56 ± 10.5	0 (0) **	2 (100)	0 (0)
	MIT group	54.5 ± 4.5	0 (0) **	21 (100)	0 (0)
Abhirami 2024 (48)	Total sample	24.6 ± 9.5	10 (43.5)	10 (100)	0 (0)
Cytotoxic agents					
Mohan 2015 (50)	Total sample	24.6 ± 2.8	NR	3 (100)	0 (0)
Bekkers 2024 (9)	Total sample	28.5 ± 3.5	9 (64.3)	10 (100)	0 (0)
Anti-glaucoma agents					
Teus 2002 (40)	Total sample	68 ± 18.6	19 (44.2)	0 (0)	0 (0)
Goldberg 2008 (12)	Latanoprost group	67.6 ± 12.4	1778 (45.2)	0 (0)	0 (0)
Alm 2008 (17)	Latanoprost/Timolol group	66.3 ± 10.9	446 (45.8)	0 (0)	0 (0)
	Adjunctive latanoprost group	65.7 ± 10.4	200 (52.6)	0 (0)	0 (0)
Teus 2002 (40)	latanoprost/timolol+ brinzolamide group	70.5 ± 12.1	9 (45)	0 (0)	0 (0)
	Dorzolamide/timolol+ latanoprost group	71.9 ± 12.8	10 (62.5)	0 (0)	0 (0)
Antimalarial agents					
Skare 2011 (5)	Antimalarial agents group	NR	10 (4.8)	69 (33)	10 (5)
Bahloul 2017 (6)	Total sample	39.2 ± 15.4	3 (7.3)	8 (66.7)	4 (33.3)
Yin 2024 (7)	Total sample	NR	31 (9.8)	79 (95.2)	4 (4.8)
Antibiotics agents					
Hanada 2016 (49)	Total sample	65.7 ± 14.5	146 (50.9)	156 (100)	0 (0)
Tang 2015 (13)	Total sample	42 ± 34.3	37 (69.8)	50 (100)	0 (0)
Immunomodulators agents					
Ah 2019 (42)	Total sample	34.4 ± 51.2	445 (40.3)	23 (100)	0 (0)
Tsilika 2013 (46)	Total sample	48.3 ± 34.8	47 (61)	9 (56.3)	7 (43.8)
Tyrosine kinase inhibitors					
Oliveira 2019 (8)	Total sample	49.3 ± 14.77	41 (55.4)	0 (0)	66 (100)
MC4R agonist					
Clément 2020 (10)	Total sample	21 ± 7.4	8 (38.1)	15 (100)	0 (0)

SD: standard deviation; n (%): number (percentage); NR: not reported; Lipo-MIT: Mitoxantrone hydrochloride liposome; MIT: Mitoxantrone Hydrochloride. * The percentage from the total patients with hyperpigmentation. ** All of the study population were females (*n* = 30).

### Quality assessment

**Figure 2:**
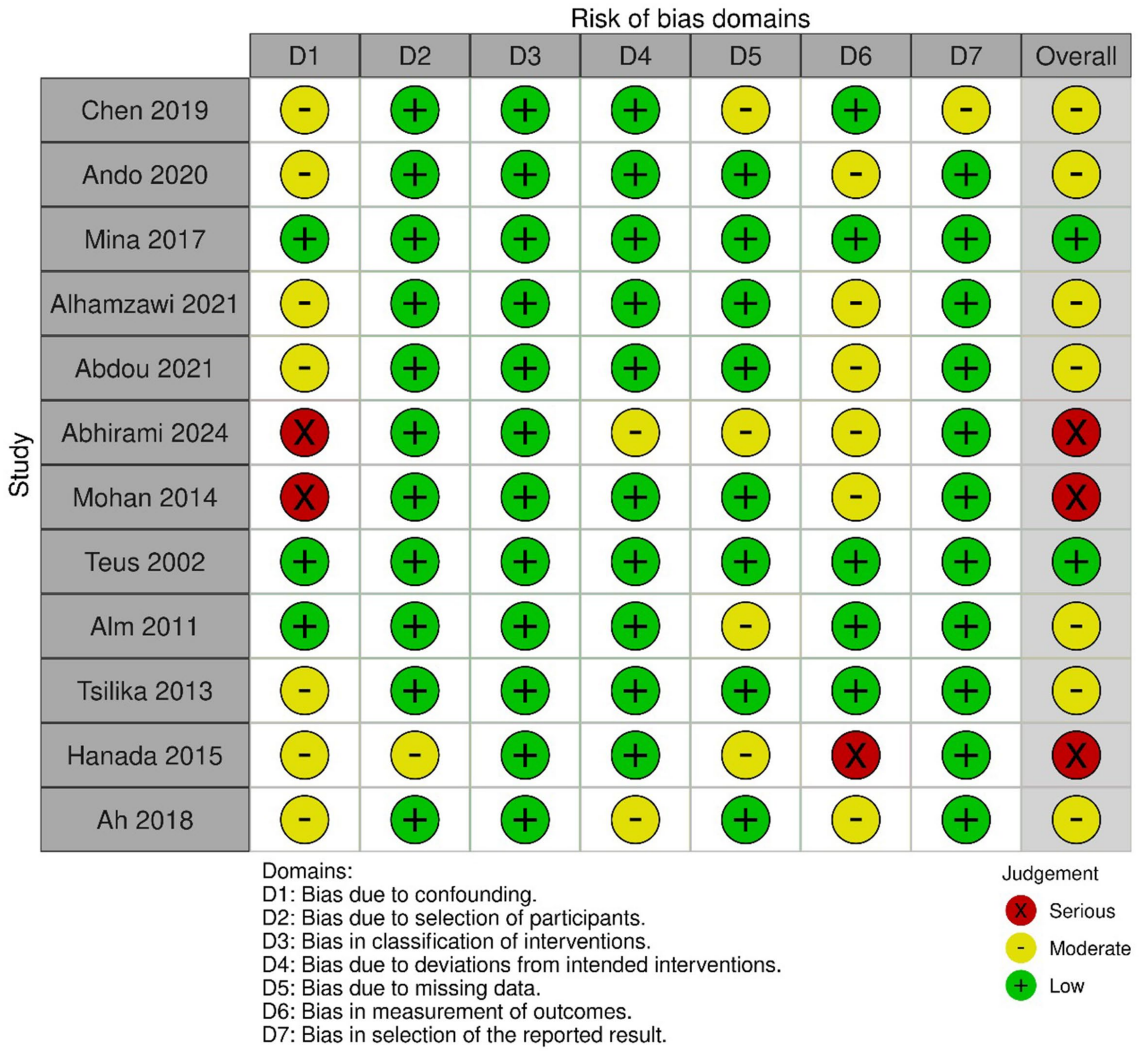
Presents the risk of bias assessment for the non-randomized trials. Summary of quality assessment of the non-randomized trials: presents the risk of bias assessment for the non-randomized trials. Mina (39) and Teus (40) were assessed to have a low overall risk of bias. Several studies, including Chen (41), Ah (42), Ando (43), Alhamzawi (44), Alm (45), Tsilika (46), and Abdou (47), were classified as having a moderate overall risk of bias. In contrast, the studies by Abhirami (48), Hanada (49), and Mohan (50) were identified as having a serious overall risk of bias, primarily due to concerns related to confounding and missing data.

The four included cross-sectional studies (5–8) demonstrated good methodological quality based on the Newcastle-Ottawa Scale (NOS) (Table 3). However, none of the studies reported the response rate (Figure 2).

**Table 3:** Quality assessment of the cross-sectional studies.

Author and year	Selection				Compara-bility	Outcome		Quality Score
	Representativeness of the sample	Sample size	Ascertainment of exposure	Non-respondents	The subjects in different outcome groups are comparable, based on the study design or analysis. Confounding factors are controlled	Assessment of outcome	Reporting the results	
Skare 2011 (5)	*	*	*		*	*	*	Good
Bahloul 2017 (6)	*	*	*		**	*	*	Good
Yin 2024 (7)	*	*	*		**	*	*	Good
Oliveria 2019 (8)	*	*	*		**	*	*	Good

* The study met that methodological quality item. ** Higher methodological quality. The total number of stars determines the overall rating (e.g., Good quality).

**Figure 3:**
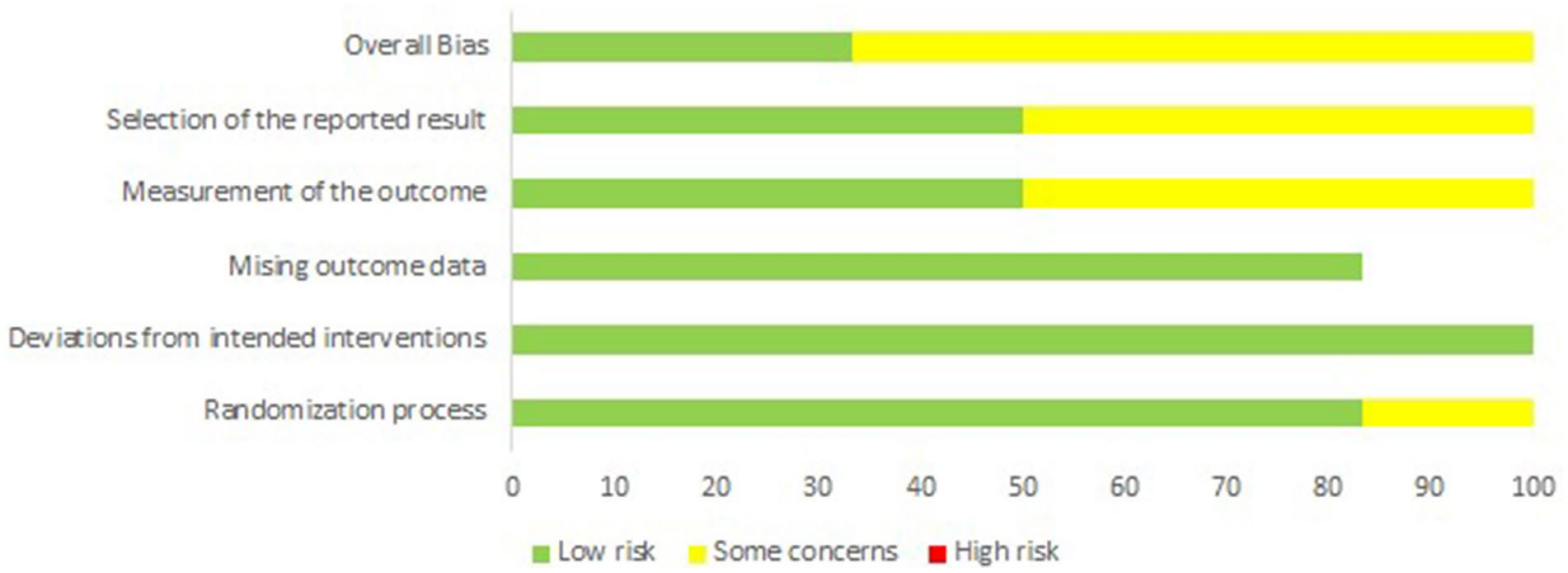
Graph summarizing the quality assessment of the RCTs.

**Figure 4:**
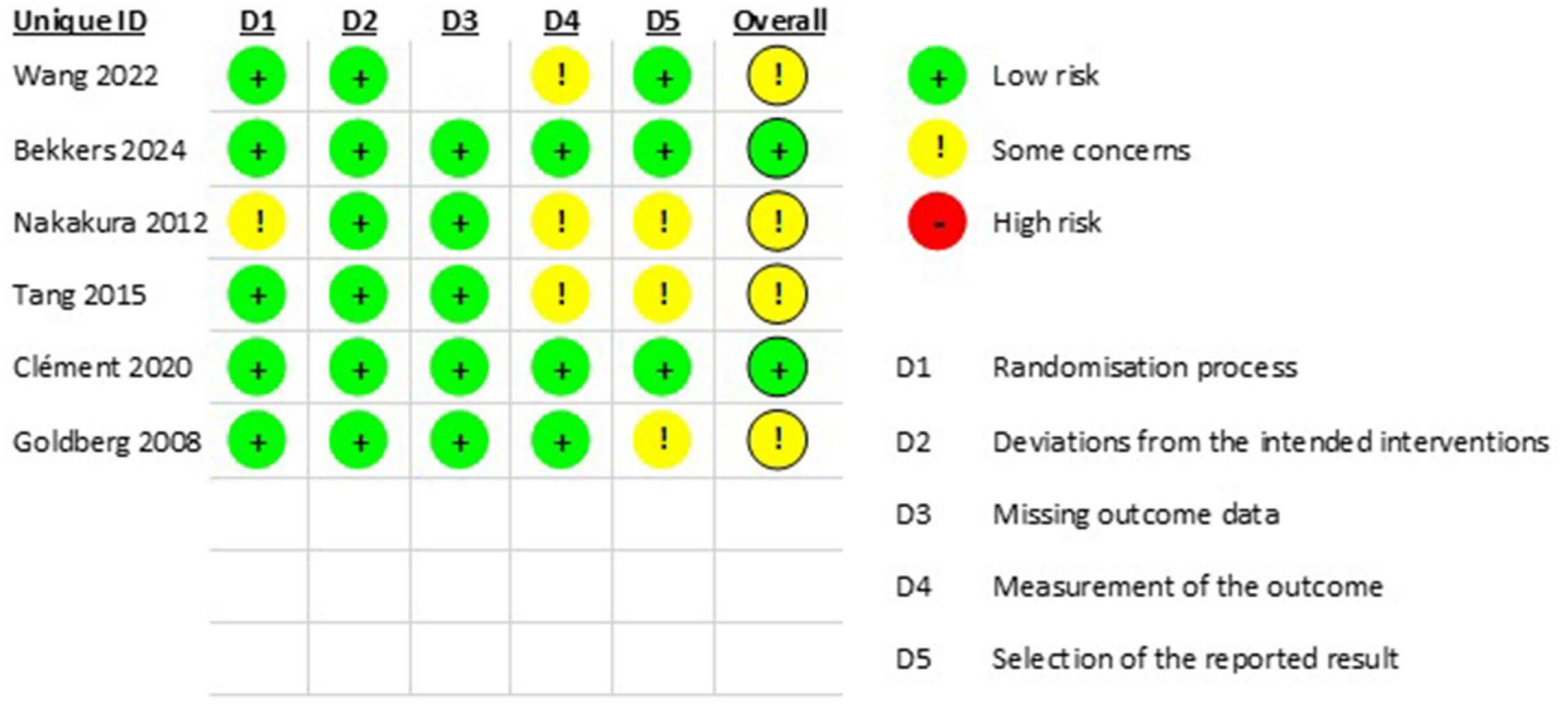
Summary of the quality assessment of the included RCTs.

Figures 3, 4 summarize the quality assessment of the included RCTs. Bekkers (9) and Clement (10) showed an overall low risk of bias (9, 10). Whereas Wang (4), Nakakura (11), Goldbreg (12), and Tang (13) showed an overall moderate risk of bias primarily due to concerns related to the randomization process, outcome measurement, or selection of reported results.

### Incidence of drug-induced hyperpigmentation across studies

**Figure 5:**
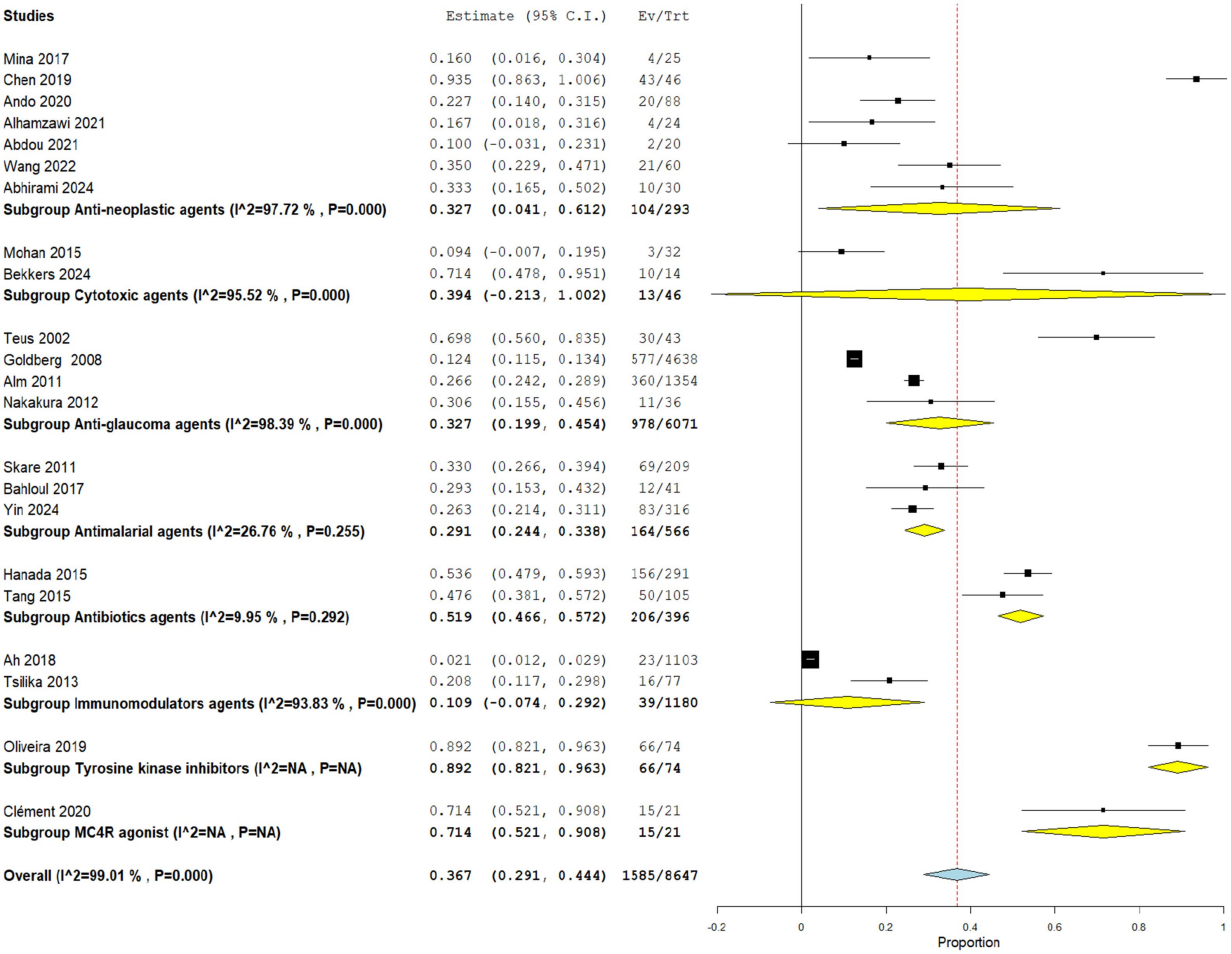
Forest plot of the drug-induced hyperpigmentation outcome.

Overall drug-induced hyperpigmentation was reported in 36.7% of the included patients. The pooled analysis shows an overall estimated proportion of 0.367 (95% CI: 0.291, 0.444) for hyperpigmentation across all studies, as shown in Figure 5.

**Figure 6:**
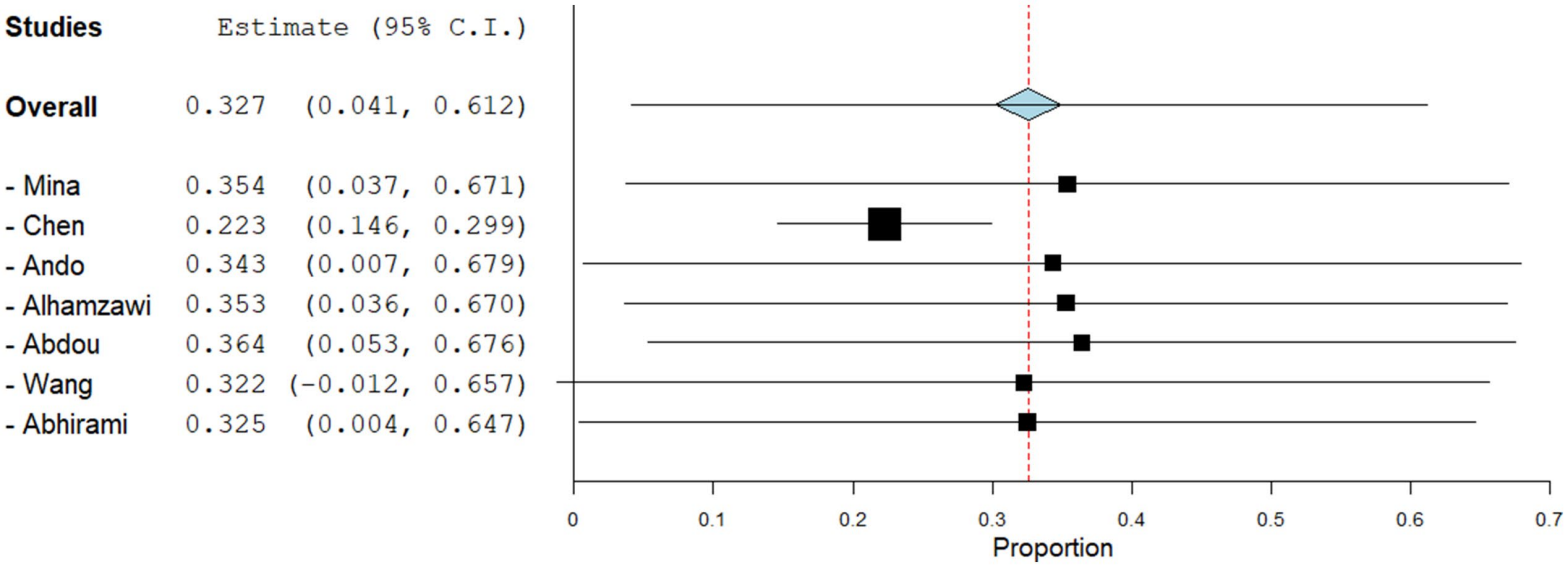
Sensitivity analysis for the studies reporting drug-induced hyperpigmentation due to anti-neoplastic agents.

Anti-neoplastic Agents induced hyperpigmentation was reported in 35.5% of patients across the 7 included studies. This subgroup demonstrated an estimated pooled proportion of 0.327 (95% CI: 0.041, 0.612) for hyperpigmentation. However, the pooled studies within this subgroup were heterogeneous (I^2 = 97.72%, *p* = 0.000). Sensitivity analysis for this subgroup is shown in Figure 6.

Cytotoxic Agent-induced hyperpigmentation was reported in 28.3% of patients across the 2 included studies. The pooled estimate for hyperpigmentation with cytotoxic agents was 0.394 (95% CI: −0.213, 1.002). However, the pooled studies within this subgroup were heterogeneous (I^2 = 95.52%, *p* = 0.000).

**Figure 7:**
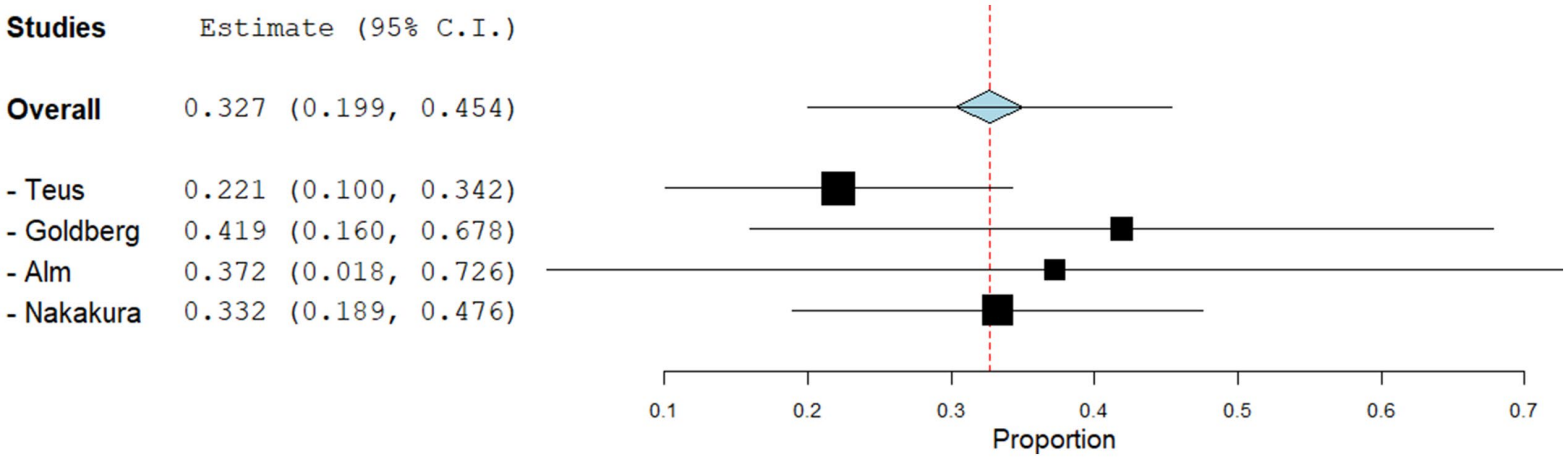
Sensitivity analysis for the studies reporting drug-induced hyperpigmentation due to anti-glaucoma agents.

Anti-glaucoma Agents induced hyperpigmentation was reported in 16.1% of patients across the 4 included studies. The pooled estimate for anti-glaucoma agents was 0.327 (95% CI: 0.199, 0.454). The pooled studies within this subgroup were heterogeneous (I^2 = 98.39%, p = 0.000). Sensitivity analysis for this subgroup is shown in Figure 7.

Antimalarial Agents induced hyperpigmentation was reported in 29.0% of patients across the 3 included studies. This subgroup showed a pooled proportion of 0.291 (95% CI: 0.244, 0.338) for hyperpigmentation. The pooled studies within this subgroup were homogenous (I^2 = 26.76%, *p* = 0.255).

Antibiotic-induced hyperpigmentation was reported in 52.0% of patients across the 2 included studies. The subgroup of antibiotics presented a pooled estimate of 0.519 (95% CI: 0.466, 0.572). The pooled studies within this subgroup were homogenous (I^2 = 9.95%, *p* = 0.292).

Immunomodulator-induced hyperpigmentation was reported in 3.3% of patients across the 2 included studies. This group demonstrated a pooled estimate of 0.109 (95% CI: −0.074, 0.292). The pooled studies within this subgroup were heterogeneous (I^2 = 93.83%, *p* = 0.000).

Tyrosine Kinase Inhibitors induced hyperpigmentation was reported in 89.2% of patients across the 1 included study. This subgroup showed a pooled estimate of 0.892 (95% CI: 0.821, 0.963).

MC4R agonist-induced hyperpigmentation was reported in 71.4% of patients across the 1 included study. This group had a pooled estimate of 0.714 (95% CI: 0.521, 0.908).

Several cases were identified for patients who experienced drug-induced hyperpigmentation. The studies, published between 2015 and 2024, originate from various countries including the US, India, Japan, Turkey, France, Canada, Morocco, Ireland, Italy, Belgium, Taiwan, Australia, Spain, Switzerland, Saudi Arabia, Brazil, Korea, and South Africa. The Patients’ age ranged from 18 to 88 years old. Several drug classes were reported, including antibiotics (most commonly minocycline), antimalarials (hydroxychloroquine), antimetabolites (hydroxyurea), antipsychotics, antidepressants, and chemotherapeutic agents. Notably, minocycline is the most frequently reported drug associated with skin pigmentation, predominantly blue-gray in color and affecting the extremities and face. Table 1 summarizes the data of these cases, including their characteristics, drug dose, duration, route of administration and hyperpigmentation data.

## Discussion

This meta-analysis investigated the incidence of drug-induced hyperpigmentation across various drug classes, revealing an overall pooled proportion of 36.7% across all included studies. This finding indicates that drug-induced hyperpigmentation is a relatively common adverse event. Previous reviews estimated that drug-induced hyperpigmentation accounts for 10–20% of all acquired pigmentation disorders (1). Krause (51), in the only prior systematic review, analyzed 306 publications and concluded that a causal relationship was strongly supported only for a limited number of drugs, such as prostaglandins, minocycline, phenothiazines, nicotine, and antimalarials (14). Our pooled incidence of 36.7% is notably higher, suggesting that DIH may be more prevalent than previously appreciated, likely due to the broader scope of pharmacovigilance and the inclusion of newer drug classes in recent studies. A unifying pattern emerges when examining drug-induced hyperpigmentation across pharmacologic classes: each class tends to produce a characteristic clinical phenotype that reflects its underlying biochemical mechanism. Cytotoxic and antineoplastic agents commonly cause pigmentary changes through direct melanocyte toxicity, vascular injury, or interface dermatitis, resulting in patterns such as serpentine supravenous hyperpigmentation or flagellate streaks. In contrast, prostaglandin analogs drive melanogenesis via tyrosinase upregulation, producing localized periocular or iris hyperpigmentation. Tyrosine kinase inhibitors and MC4R agonists demonstrate class-specific effects linked to altered melanocyte signaling or metabolite deposition, manifesting as diffuse yellow-gray or mucosal discoloration. Antibiotics such as minocycline show a spectrum of phenotypes—blue-gray macules, muddy-brown patches, or scar-associated pigmentation—corresponding to mechanisms that include iron chelation, drug-metabolite deposition, or increased melanin synthesis. Similarly, agents associated with photosensitivity (e.g., amiodarone, thiazides, fluoroquinolones) induce pigmentation through phototoxic or photoallergic pathways that culminate in post-inflammatory melanosis. This continuum—pharmacologic class → mechanism of pigmentation → clinical phenotype—provides a framework that enhances diagnostic accuracy and strengthens causal attribution in suspected cases of drug-induced hyperpigmentation.

Our subgroup analysis revealed substantial variability in the incidence of drug-induced hyperpigmentation across different classes. The incidence among patients using anti-neoplastic agents was 35.5%. Whereas cytotoxic agents induced hyperpigmentation has an incidence of 28.3%. Intravenous 5-fluorouracil, an anti-neoplastic agent, is associated with several patterns of hyperpigmentation (15–17). These include pigmented macules of the palms, soles, nails, and oral mucosa or serpentine supravenous pigmentation, which appears as a pigmented band along the course of a vein. The mechanism of 5-fluorouracil-induced hyperpigmentation is thought to be due to the cytotoxic effects of the drug damaging blood vessels and causing an interface dermatitis with secondary pigment dropout (15–17). Bleomycin, a cytotoxic drug, is well-known for causing *flagellate* hyperpigmentation which presents as erythematous linear streaks especially over bony prominences (18). This hyperpigmentation is thought to be due to trauma, with scratching and leakage of bleomycin into the skin where it is not properly metabolized and leading to subsequent melanogenesis (19).

Anti-glaucoma agents presented with an incidence of 16.1%. Prostaglandin analogs, such as bimatoprost and latanoprost, are known to stimulate melanogenesis through stimulating the activity of tyrosinase and increase the dendritic structure of melanocytes and melanin synthesis (20). This can result in hyperpigmentation of the eyelids and periorbital area with direct application (21–23) and permanent pigmentation of the iris when used for glaucoma (24).

The highest incidence for hyperpigmentation in our meta-analysis was identified among Tyrosine Kinase Inhibitors and MC4R agonists with an incidence of 89.2 and 71.4%, respectively. Imatinib, a tyrosine kinase inhibitor, has been shown to induce hyperpigmentation of the skin and oral mucosa, including greyish discoloration of the hard palate in nearly 90% of patients in one study (8, 25). While the precise mechanism is unclear, a paradoxical effect on c-kit-stimulating melanogenesis is hypothesized (26). Similarly, sunitinib also leads to hyperpigmentation, specifically yellowing of skin and hair thought to be due to deposition of sunitinib metabolites (25, 27). We found that the incidence of Antibiotic-induced hyperpigmentation is about 52%. Minocycline, a well-documented cause of hyperpigmentation, presents in four types: Type I (blue-black in scars/inflammation), Type II (blue-gray on clear skin, shins/forearms), Type III (muddy-brown in sun-exposed areas), and Type IV (similar to Type I but on back scars) (28, 29). These variations are due to differing mechanisms, including iron chelation, metabolite deposition, and increased melanin production (17). Clofazimine causes a red-blue skin hue that deepens to red-brown due to metabolite deposition (13, 30, 31).

Beyond minocycline, multiple other medications are documented to cause photo-induced pigmentary changes, making photosensitivity an important mechanism in drug-induced hyperpigmentation. Drugs such as amiodarone, hydrochlorothiazide, fluoroquinolones, doxycycline, and certain BRAF or tyrosine kinase inhibitors have been implicated in phototoxic or photoallergic reactions that can evolve into hyperpigmentation (14, 32). In phototoxic reactions, the drug or its metabolite absorbs ultraviolet radiation, predominantly UVA, leading to reactive oxygen species generation, direct cellular injury, and enhanced melanogenesis, whereas photoallergic reactions involve hapten formation and delayed hypersensitivity with secondary pigment deposition (33). Lozzi et al. emphasized that photo-induced cutaneous eruptions remain among the most frequent drug-related adverse events, underscoring the importance of recognizing photosensitizing agents and implementing photoprotective measures during therapy (34).

To better predict drug photosensitizing potential, prevent and manage cutaneous adverse events, and guide alternative therapy, targeted investigations can be considered. As summarized in recent reviews, phototesting (to define the UVA/UVB action spectrum) and photopatch testing (when a photoallergic mechanism is suspected) are useful adjuncts in establishing causality and characterizing the reaction, with rechallenge evidence reported for selected agents (14, 32–34).

In addition, histopathology may assist in differentiating phototoxic from photoallergic patterns when clinical features overlap (14). These investigations, coupled with counseling on sun-protective measures and drug substitution where warranted, may improve recognition and clinical decision-making in suspected drug-related photosensitivity (14, 32–34).

We found that the incidence of Anti-malarial-induced hyperpigmentation is 29%. This often manifests as blue-gray macules, primarily affecting the anterior legs, but also sometimes the head, and can also affect mucosa (35). The underlying mechanism may involve the formation of drug-melanin complexes and dermal vessel damage (36, 37), with evidence of both melanin and hemosiderin deposition in affected areas (38). In most of these classes, we observed heterogeneity among the included studies, mainly due to differences in the baseline and characteristics of the included populations, dosages, routes of administration, and durations of treatment. Conversely, other drug classes presented homogeneity among the included studies, such as antimalarial and antibiotic agents.

This meta-analysis provides clinicians with valuable information on the incidence of hyperpigmentation across several drug classes. The study highlights the importance of monitoring patients for hyperpigmentation and considering alternative treatment options when appropriate.

Identifying a clear connection between a drug and its pigmentary effect can be challenging, as the onset of pigmentation often occurs gradually or long after treatment begins, which can obscure the causal link. This challenge becomes greater in patients using multiple medications or with pre-existing skin conditions. A thorough review of the treatment timeline, distribution of pigmentation, and other contributing factors is therefore essential. When available, histopathological findings or improvement after discontinuation of the suspected agent can help confirm the relationship. Strengthening this clinical link is important to improve recognition, prevent unnecessary drug withdrawal, and guide patient counseling.

Future research should focus on exploring the mechanisms of drug-induced hyperpigmentation, identifying at-risk patient populations, and developing strategies for prevention and treatment. The meta-analysis builds upon existing literature by providing a quantitative estimate of the overall incidence of drug-induced hyperpigmentation and performing a sub-group analysis of different drug classes, which was not available in previous literature. However, we acknowledge that there are some limitations. Firstly, we included both randomized and non-randomized controlled trials. Secondly, there was a significant level of heterogeneity in some of the pooled groups. Future research should seek to identify factors that account for this heterogeneity, such as study design, patient characteristics, specific drugs, and dosages. It should also consider different routes of administration. Further high-quality trials of drug-induced hyperpigmentation are needed.

### Limitations

This review has several limitations. First, by excluding case reports, case series, and narrative reviews, we may have overlooked medications with frequent associations with drug-induced hyperpigmentation (DIH) that are primarily documented in such reports. While this decision enhanced methodological rigor and minimized potential bias, it may have limited the comprehensiveness of our findings. Second, heterogeneity across included studies was notable, reflecting variations in study design, patient populations, diagnostic criteria, and reporting practices. Third, the number of eligible studies for certain drug classes was small, which may have led to imprecise pooled estimates. Finally, publication bias cannot be excluded, as studies with negative or less striking findings are less likely to be published. These factors should be considered when interpreting the results, and future research including larger, standardized, and multicenter investigations is warranted.

## Conclusion

This meta-analysis reveals a significant overall incidence of drug-induced hyperpigmentation at 36.7% across all included studies. However, our subgroup analysis identified substantial variation across drug classes. Notably, tyrosine kinase inhibitors and anti-neoplastic agents exhibited higher incidences, while anti-glaucoma agents and immunomodulators showed a lower incidence. These results highlight the importance of considering drug class when assessing the risk of hyperpigmentation. Further research should explore these differential risks and their underlying mechanisms.

## Data Availability

The original contributions presented in the study are included in the article/supplementary material, further inquiries can be directed to the corresponding author.
